# eLearning course for improving civil registration and vital statistics systems

**DOI:** 10.1186/s41043-019-0182-4

**Published:** 2019-10-18

**Authors:** Samuel Mills, Sheila Jagannathan, Jane Kim Lee, Bahie Mary Rassekh

**Affiliations:** World Bank Group, 1818 H Street, NW Washington, DC 20433 USA

**Keywords:** Civil registration and vital statistics, Capacity building, eLearning

## Abstract

The World Bank Group (WBG), in partnership with the Global Civil Registration and Vital Statistics (CRVS) Group, the Korea Ministry of Economy and Finance, and the WBG Open Learning Campus, launched the first comprehensive CRVS eLearning course in May 2017. The development of this course demonstrates the commitment and collaboration of development partners and governments working closely together in building the capacity of national institutions to improve CRVS systems in low- and middle-income countries. As of December 2018, over 2300 learners from 137 countries have enrolled in the course. This paper discusses how the course has been developed, disseminated, and evaluated thus far. It also presents the challenges faced and how the course has improved based on feedback from course participants.

## Developing the CRVS eLearning course

One of the constraints identified in the *Global Civil Registration and Vital Statistics (CRVS) Scaling Up Investment Plan 2015–2024* [1] is limited technical capacity within national institutions in low- and middle-income countries for improving CRVS systems. Yet, no academic institutions offer degree programs that cover the various components of CRVS. To help fill this gap, in January 2016 the World Bank Group (WBG) developed a concept note and initiated consultations with members of the Global CRVS Group [2] (which comprises organizations that support CRVS systems at the global, regional, or national levels) and with academic institutions on a proposal for developing a state-of-the-art CRVS eLearning course. Subsequently, the WBG worked closely with international technical experts from various institutions (including the United States National Center for Health Statistics which had developed a training course on CRVS geared towards producing vital statistics [3]) to develop draft modules of the course. The WBG and the Centre of Excellence for CRVS cohosted an expert group workshop in Ottawa, Canada, from July 11 to 14, 2016, to review and fine-tune the draft modules. More than 50 experts, including those from academia, international and regional institutions, and national civil registration agencies, joined the workshop. The WBG team then updated the modules based on feedback received at the workshop, and international experts virtually reviewed the updated contents through December 2016. With guidance from the manager of the WBG Open Learning Campus (OLC), the WBG team packaged the technical content into PowerPoint storyboards from October 2016 to March 2017. The OLC Team used the storyboards to turn the eLearning modules into multimedia videos with narration and interactive exercises. The OLC Team carried out the multimedia production and pretesting from March to May 2017. Each module was accompanied by an introductory video featuring international experts briefly sharing what each module covered; these introductory video productions were carried out from February to April 2017.

Under the auspices of the Global CRVS Group, the WBG partnered with the Korea Ministry of Economy and Finance and the OLC to launch the first comprehensive CRVS eLearning course on May 23, 2017, at a high-level event in Seoul, Republic of Korea. The development of this course demonstrates the commitment of development partners and governments working closely together to achieving the Sustainable Development Goal Target 16.9, “By 2030, provide legal identity for all, including birth registration.”

The OLC has created a digital environment where all types of learning are integrated, and all development stakeholders—WBG staff, clients, partners, and the public—can optimize their learning opportunities. The platform, which launched in January 2016, integrates innovations in learning through multimedia, data, gamification, and augmented and virtual reality. The OLC offers flexible paths to learning across its three “schools”: WBa Academy for formal, deeper dives into development topics through self-paced and facilitated online courses; WBc Connect for a more informal exchanging of ideas and knowledge through communities of practice; and WBx Talks for a mix of bite-sized learning opportunities that provide a quick overview or insight on development topics.

## Content and format of the CRVS eLearning course

The course aims to provide practical tools and approaches in building and maintaining state-of-the-art CRVS systems that are linked to identity management systems and tailored to local contexts, and which eventually can contribute to alleviating poverty and promoting shared prosperity. The primary target audiences are policy-makers, personnel working on civil registration and identification systems, health care workers, and officials in national statistics offices. The secondary target audiences are university students interested in pursuing a career in institutions that are part of the CRVS system, researchers, development practitioners, and civil society organizations.

The comprehensive eLearning course is presented in 13 modules:

- Module 1. Importance of CRVS Systems
- Module 2. Overview of CRVS Systems
- Module 3. Institutional Arrangements of CRVS Systems
- Module 4. Legal Frameworks of CRVS Systems
- Module 5. Birth Registration and Adoption
- Module 6. Death Registration
- Module 7. Marriage and Divorce Registration
- Module 8. Analysis and Use of Vital Statistics
- Module 9. Presentation and Dissemination of Vital Statistics
- Module 10. CRVS Digitization
- Module 11. CRVS Assessment and Strategic Planning
- Module 12. Identity Management Systems
- Module 13. Refugees, Internally Displaced Persons, Stateless Persons, and CRVS Systems

Each module comprises 1–10 lessons and each lesson presents the following: key messages on certain aspects of the CRVS system; country examples that highlight best practices from around the world; self-tests and other interactive exercises to check the learner’s understanding; and a downloadable, full PDF version of the content.

The eLearning course is delivered into two formats: self-paced [4] and virtually facilitated [5]. The self-paced format, which opened for enrollment on May 23, 2017, is offered for busy professionals who are able to take the course at their own pace and finish at any time. It can be accessed at the following link: https://olc.worldbank.org/content/civil-registration-and-vital-statistics-systems-self-paced. Learners receive a basic level certificate after successfully completing core modules 1–3 and can subsequently enroll in the facilitated format to receive an Advanced Level Certificate. The facilitated (Advanced Level) course can be accessed at https://olc.worldbank.org/content/civil-registration-and-vital-statistics-systems-advanced-level-facilitated. The first virtually facilitated course was offered from September 11, 2017 to October 20, 2017, and the fourth class ended in February 2019. The French and Spanish versions of the self-paced course were released on June 20, 2018 and October 12, 2018 respectively.

In addition to the online version, the print pdf versions of the course are available for countries or trainers to adapt for face-to-face trainings. For instance, the materials were used for training of trainers (ToT) for representatives from English-speaking African countries in Namibia in April 2018, and a similar regional ToT was held in Senegal for representatives from French-speaking countries in Africa. Following the regional ToT, the Namibia Ministry of Home Affairs and Immigration used the materials to organize a training workshop for civil registration and identification personnel.

## Dissemination

The course has been widely disseminated via email to CRVS global, regional, and national networks, as well as via blogs, social media, and at several national and international conferences. To incentivize learners, at the Fourth Conference of African Ministers Responsible for Civil Registration (December 2017, Nouakchott), awards were given to Ethiopia and Namibia for having the most learners who completed the basic level self-paced CRVS course. Similarly, at the third meeting of the Regional Steering Group for CRVS in Asia and the Pacific (October 2017, Bangkok), awards were given to the Philippines and Myanmar. At the Latin America and Caribbean Civil Registrars CLARCIEV meeting (October 2018, Cartagena, Colombia), the Spanish version of the course was presented, and awards will be provided at its 2019 annual meeting.

## Course participants

**Fig. 1:**
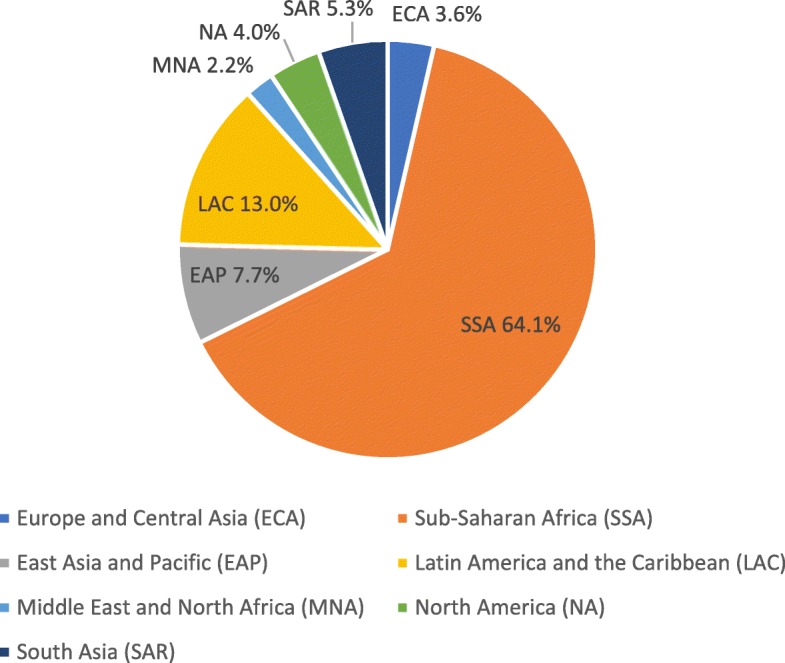
Geographic region of learners in the civil registration and vital statistics courses

During the first year and a half of the course, over 2300 learners enrolled in the self-paced course, including more than 500 participants in the French and Spanish versions of the course. Of all the learners, approximately two-thirds were from Sub-Saharan Africa, 13% from Latin America and the Caribbean, and 8% from East Asia and the Pacific (see Fig. 1). The countries with the greatest number of participants, in descending order, were: Cameroon (most number of participants), Lesotho, Ethiopia, Namibia, Mexico, the United States, Rwanda, India, Philippines, Grenada, and Myanmar.

Learners joined from 136 countries representing all regions, including 40 countries in Sub-Saharan Africa, 25 countries in Europe and Central Asia, 23 countries from Latin America and the Caribbean, 22 countries from East Asia and the Pacific, 15 countries from the Middle East and North Africa, 8 countries from South Asia, and 3 countries from North America (see Tables 1 and 2).

**Table 1:** Geographic region of countries represented by learners in the civil registration and vital statistics courses

Region	Sub-Saharan Africa	Europe and Central Asia	Latin America and the Caribbean	East Asia and the Pacific	Middle East and North Africa	South Asia	North America	Total
Number of countries	40	25	23	22	15	8	3	136
Percent of total	29.4	18.4	16.9	16.2	11.0	5.9	2.2	100

**Table 2:** Income level of countries represented by learners in the civil registration and vital statistics courses

Country income level	Low income	Lower-middle-income	Upper-middle-income	High-income	Total
Number of countries	32	34	38	32	136
Percent of total	23.5	25.0	27.9	23.5	100

The large majority of learners worked at the government/regulatory agency of their respective countries (54.8%) as well as professional or technical staff/officers (35.3%). The remaining 10% of participants worked in the following organization type or capacity: bilateral or multilateral agency (3.2%), academic/training institution or research institute (2.9%), service/utility provider (1.1%), not-for-profit non-governmental organization (1.1%), private sector organization (0.7%), law enforcement or traditional authority (0.4%), association (0.3%), youth group (0.1%), political party (0.1%), or labor/trade union (0.1%).

**Fig. 2:**
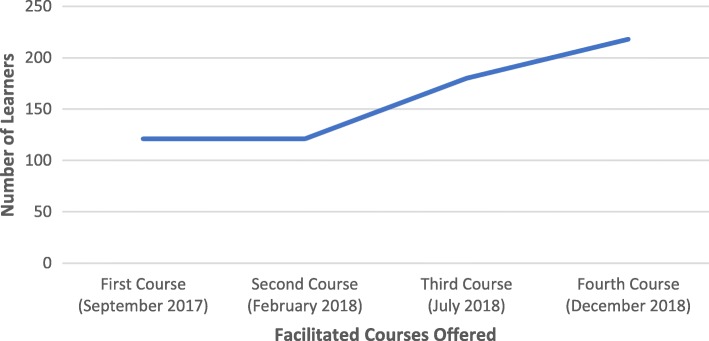
Number of learners enrolled in the four facilitated CRVS courses

Of the 1403 learners who completed the self-paced course, 640 enrolled in the facilitated course and 242 completed the facilitated course (see Fig. 2).

Learners for the facilitated course completed an anonymous self-evaluation form, and the data were collated by a third party, the World Bank Knowledge Management. Participants answered a range of questions, such as how the course could be improved and how (if any) the course has assisted them in their work. Evaluation findings have been overwhelmingly positive so far. For instance, on a scale of 1 to 7 (with 7 being the highest), 63% rated the overall usefulness of the course at 7 and 23% rated it at 6; similarly, 76% rated at 7 whether they would recommend the course to their colleagues and 13% rated this question at 6. Participants further indicated how they planned to use the skills and knowledge gained from this course, many citing that they would use it to strengthen the CRVS systems in their own countries.

In terms of participants’ responses regarding how the course assisted them in their work, a great deal of positive feedback was received. Participants from various country governments, including ministries, national identification agencies, and civil registration agencies, among others, said the following regarding the course (identifying information excluded):

“I realized that this is what we are doing in our everyday work at the National Identification Agency in (-- country). We are in the process of modernizing CRVS from a paper-based one to a digitized one… It's an interesting course!”

“The course has been valuable to enhancing my knowledge in the different aspects of CRVS (from legal framework to the different Vital Events, to Digitization, and also Integration issues with ID and other sector systems like in health, economic, etc.), and thereby to be able to work easily and closely with the technical personnel working on CRVS.”

“With my current job position, the course has helped me gain a lot of insight on international matters relating to CRVS, which otherwise would not have been possible. This knowledge will be much useful when I have to work on related projects.”

“I plan on training doctors on the Medical Certificate of Causes of Death and training doctors on the introduced e-death notification system.”

“I will use the knowledge to improve the CRVS in my country especially on vital statistics report writing which is rarely done.”

“I am planning to share the knowledge I have gained with my colleagues and also want to engage my counterparts at the Ministry of Health and the Ministry of Security to give them information especially when it comes to establishment of cause of death.”

“I will use it as it is providing all necessary materials and tools that I always miss to improve my usual work (as the head of vital statistics division).”

“I am going to train my colleagues, recommend stakeholders to take this course, train stakeholders, sensitize Home Affairs Ministry management to improve the civil registration in the country, urge the country to do CRVS assessment and actually participate in the CRVS assessment and do everything possible to improve the state of CRVS in my country.”

There were also challenges that learners stated in their course evaluation. Based on evaluation results, the greatest challenge for learners was related to ease of access to the content. For all four facilitated course cohorts, the question for evaluation, “the content was easy to access” consistently received the weakest results. It improved over time but remains an area of challenge for the course (see Table 3).

**Table 3:** Evaluation from the facilitated course regarding ease of access to the content

	Participants’ aggregated responses evaluation question: “the content was easy to access” (percent of respondents)						
Facilitated course cohort number	1^a^	2	3	4	5	6	7
Cohort 1	3	3	5	8	33	18	31
Cohort 2	0	4	4	0	27	35	31
Cohort 3	0	0	2	7	15	31	45
Cohort 4	0	0	0	8	25	35	33

^a^*1*=strongly disagree; *7*=strongly agree

Additional stated challenges included language difficulties for non-English speakers, requests for the course to be available to learners for a longer duration, to improve the platform and course navigation, and to provide faster and more feedback on participants’ completed assignments and eDiscussion submissions.

## Improving the course based on feedback from learners

Based on course participants’ feedback, refinements were made to the course to strengthen both content and delivery. Regarding the content, the authors of each module updated their print versions at least once a year, some more often, which the WBG Team then repackaged into storyboards and the OLC Team incorporated into multimedia videos. These updates ranged from making minor edits (such as adding updated hyperlinks to online resources) to making new lessons to cover more topics that were suggested by the learners.

In terms of how the course delivery could be improved, some learners for whom English is not a native language reported the language barrier as a challenge. The course was launched in English only. It is now available in several languages; the basic course is available in French, Spanish, and English, and it is being translated into other languages, including Bahasa Indonesia and the Lao language.

Another challenge related to accessing the course online. The online delivery of the course through the WBG’s OLC website made it possible for anyone with Internet access to be able to take the course free of charge. The basic course is available at any time for learners and the facilitated course is available regularly throughout the year. While the downloadable print version provides the entirety of both courses to learners, the multimedia video format with narration and interactive exercises provides the main content delivery. This delivery method made learning more engaging for the vast majority of participants. However, for some people who live in areas with limited or slow Internet connectivity, the multimedia videos have at times been difficult to access. In response, the WBG team created a frequently asked questions (FAQ) document after the first facilitated course, which includes step-by-step instructions on resolving common Internet browser-related issues, how to navigate the course website, and contact information for information and technology experts at the OLC, among others. The course is also available on personal computers, smartphones, and tablets.

The facilitated course’s length of time has also been adjusted, along with the requirements for course completion. One of the main advantages of the facilitated format is that it offers participants the opportunity to ask questions and have their written assignments answered and evaluated by various international CRVS experts (instructors), most of whom are authors of the various 13 modules of the course. Through the eDiscussion forum, learners can interact with the instructors and other course participants, fostering a rich sharing of country experiences, lessons learned, and helpful resources. This unique opportunity for learners to directly communicate with many CRVS experts in one forum is possible because some CRVS experts volunteer to serve as instructors of the course while others are paid by the WBG. The contributions of these CRVS experts are greatly appreciated and will continue to be needed to carry forward the facilitated format of the course.

The type of feedback received by participants has also changed over time. Initially, there was more feedback regarding evaluators providing faster and more feedback on assignments, whereas that decreased by 33% between the first and second cohort, 50% between the second and third cohort and was only provided by a couple of participants by the third and fourth cohorts. By the fourth cohort of participants, two-thirds of respondents explicitly said when asked about the least favorite part of the course that there was nothing. The most commonly cited recommendations for the facilitated course’s fourth cohort included providing the course in other languages (similar to the self-paced course which is available in three languages now) and providing more time for participants to complete the course by take internet connectivity into account.

## Conclusion

A large number of people from several countries are taking this course and improving their ability to translate what they learn into stronger CRVS systems in their countries. As these participants have commented after taking the course, this course has enabled them to build their own capacities to apply the course principals in their own work and to further train others in the knowledge and skills gained. Although there have been challenges in the course, refinements are continually being made to the course to strengthen both content and delivery especially taking course participants’ feedback into account. Those who developed the course continue to make efforts to improve its content and delivery as well as increase its availability, in order to contribute to strengthening the capacity of CRVS systems globally. To that end, the course content is continuously updated, and the organizers of the course seek and welcome more CRVS experts to serve as instructors of the course to virtually facilitate fruitful dialogues and knowledge sharing among the course learners and practitioners.

## Data Availability

Not applicable

## Abbreviations

CRVS: Civil registration and vital statistics
FAQ: Frequently asked questions
IDM: Identity management
OLC: Open Learning Campus
ToT: Training of trainers
WBG: World Bank Group
